# Investigating the influence of an adjustable zoned air mattress on sleep: a multinight polysomnography study

**DOI:** 10.3389/fnins.2023.1160805

**Published:** 2023-04-20

**Authors:** Yu Wei, Yongpeng Zhu, Yihan Zhou, Xiaokang Yu, Huiping Lin, Lijun Ruan, Hua Lei, Yuxi Luo

**Affiliations:** ^1^School of Biomedical Engineering, Shenzhen Campus of Sun Yat-sen University, Shenzhen, China; ^2^De Rucci Healthy Sleep Limited Company, Dongguan, Guangdong, China; ^3^Key Laboratory of Sensing Technology and Biomedical Instruments of Guangdong Province, Shenzhen Campus of Sun Yat-sen University, Shenzhen, China

**Keywords:** sex difference, air mattress, sleep quality, sleep architecture, multi-night sleep study

## Abstract

**Introduction:**

A comfortable mattress should improve sleep quality. In this study, we sought to investigate the specific sleep parameters that could be affected by a mattress and explore any potential differences between the effects felt by each sex.

**Methods:**

A total of 20 healthy young adults (10 females and 20 males; 22.10 ± 1.25 years) participated in the experiments. A smart adjustable zoned air mattress was designed to maintain comfortable support, and an ordinary mattress was used for comparison. The participants individually spent four nights on these two mattresses in four orders for polysomnography (PSG) scoring. Sleep architecture, electroencephalogram (EEG) spectrum, and heart rate variability (HRV), which reflect the central and autonomic nervous activities, were used to compare the difference between the two mattresses.

**Results:**

An individual difference exited in sleep performance. The modes of influence of the mattresses were different between the sexes. The adjustable air mattress and the increase in experimental nights improved female participants' sleep efficiency, while male participants exhibited a smaller response to different mattresses. With an increasing number of experiment nights, both sexes showed increased REM and decreased N2 proportions; the N3 sleep proportion decreased in the male participants, and the heart rate decreased in both sexes. The performance of the EEG spectrum supports the above results. In addition, the adjustable air mattress weakened automatic nerve activity during N3 sleep in most participants. The female participants appeared to be more sensitive to mattresses. Experiment night was associated with psychological factors. There were differences in the results for this influence between the sexes.

**Conclusion:**

This study may shed some light on the differences between the ideal sleep environment of each sex.

## 1. Introduction

Sleep is a highly conserved innate behavior for all animals, including humans, and it plays an essential role in many behavioral and physiological functions, including immunity (Zielinski and Krueger, 2011; Zielinski et al., 2016; Miletinova and Buskova, 2021), growth, cognition (Scullin and Bliwise, 2015), emotional control (Tempesta et al., 2018), memory consolidation (Rasch and Born, 2013), and brain metabolite clearance (Xie et al., 2013). A lack of sleep causes hormonal disorders, obesity, diabetes, and cardiovascular diseases and increases the risk of Alzheimer's disease (Holth et al., 2019). Nevertheless, obtaining good sleep is considered extravagant for adults living in modern society, with a global percentage of 19.22% achieving this (Robbins et al., 2020).

Mattresses are indisputably an important factor in sleep quality (Taylor and Gradworks, 2015). According to the study of Park et al., a comfortable mattress can be evaluated by spinal curvature, body pressure distribution, and subject ratings. The firmness of mattresses and anthropometric features should also be considered (Park et al., 2001). A mattress that can support the spine's curvature can naturally increase sleep efficiency and the proportion of deep sleep and reduce unnecessary body movements (Lee and Park, 2006). Manual muscle testing was also used to choose a mattress. Kuo et al. (2013) reported that a strong bedding system would decrease cardiovascular sympathetic modulation and increase cardiac vagal activity and baroreceptor reflex sensitivity during sleep. Some studies have also revealed that medium-firm bedding provides benefits for patients with chronic low back pain (Kovacs et al., 2003; Jacobson et al., 2006, 2009).

In order to designed as ergonomically correct as possible, zoned mattresses with variable rigidity have grown in popularity both in application and research. Such zoned mattresses are commonly divided into ~3–10 parts according to the body contours, and the influence of zoned mattresses on sleep tends to vary by group. Varying the support pressure of the shoulder and hip of the mattress would improve sleep (Baek et al., 2022), especially for poor sleepers (Yu et al., 2020). A previous study using a zoned spring mattress (Verhaert et al., 2011) indicated that suitable support improved the sleep quality for the participants who spent most of the time in a lateral or prone posture. Zhang et al. (2021) recently proposed a smart mattress that could automatically recognize the sleeping position and adjust the pressure during sleep, but it lacked the support of sleep experiments. A smart, real-time adjustable zoned air mattress was designed as part of this study. The main functions of this mattress are as follows: (1) assessing the sleeper's body type by evaluating pressure distribution and (2) monitoring the sleeping posture and adjusting the pressure in three zones to provide suitable support throughout the night.

Researchers have reported that there are differences in the choice of sleep environment and mattress between men and women. In the study of Bjorvatn et al. (2017), The pillow was considered most important for sleep, closely followed by the mattress, but also the comforter was rated as very important by one out of four females and one out of six males (Table 1). Females and older adults rated the importance as higher compared to males and younger adults, respectively. In the paper of Hu et al. (2019), Results indicated that the back pressure and waist pressure were different with genders.

**Table 1:** Demographic characteristics of the participants.

**Variables**	**Female**	**Male**	***p*-value**
Num	10	10	/
Age	22.20 ± 1.33	22.00 ± 1.23	0.725
BMI (kg/m^2^)	21.24 ± 5.14	20.46 ± 1.73	0.597
*S* (m^2^)	1.68 ± 0.12	1.83 ± 0.08	0.010^**^
PQSI	6.00 ± 4.76	5.60 ± 3.63	0.909

The data in the table are mean ± standard deviation.

BMI, body mass index; S, mean body surface; PQSI, Pittsburgh Sleep Quality Index.

S = 0.0061 × height + 0. 0124 × weight – 0. 0099.

Significance codes: ^**^p < 0.01.

In addition to the traditional sleep architecture analysis, the power spectrum distribution of EEG and HRV indexes were also evaluated in this study for a more detailed comparison. According to the American Academy of Sleep Medicine (AASM) guidelines (Berry et al., 2012), sleep scoring is mainly dependent on the specific waveforms of EEG signals, and such waveforms correspond to the activities of different frequency bands. An increase in delta and theta band power was associated with sleep deepening (Svetnik et al., 2017; Gao et al., 2020a; Long et al., 2021). Delta power is also considered to reflect sleep pressure (Leger et al., 2018). HRV is the effective index for ANS activity (Jarvelin-Pasanen et al., 2013). The lower frequency (LF) and high frequency (HF) bands' power reflect the activity of the sympathetic nervous system and parasympathetic nervous system, respectively, and the ratio of LF to HF embodies the balance between them (Otzenberger et al., 1998; Stein and Pu, 2012; Hsu et al., 2021).

In this study, we attempted to systematically investigate the impact of a smart air mattress designed during the course of the study, especially regarding which sleep parameters can be affected by mattresses. Psychological factors and personal preferences should be important factors in mattress selection. Thus, individual- and sex-based differences are also important parts of this study.

## 2. Methods

### 2.1. Participants

A well-defined group of participants, consisting of 20 healthy undergraduate and graduate students, was recruited in this study, and their physical characteristics are illustrated in Table 1. All participants gave written informed consent to participate. We relied on questionnaires and interviews to determine whether they met the inclusion and exclusion criteria. The inclusion criteria were right-handed adults with a medium body type, regular sleep patterns, and good physical and mental health. The exclusion criteria were people with any history of epilepsy, insomnia, depression, or other medical problems that can interfere with normal sleep or those with any history of neck, back, lumbar, or leg pain. This study was approved by the Ethics Committee of Guangdong 999 Brain Hospital (approval number: 2020-010-059).

### 2.2. Mattress properties

**Figure 1:**
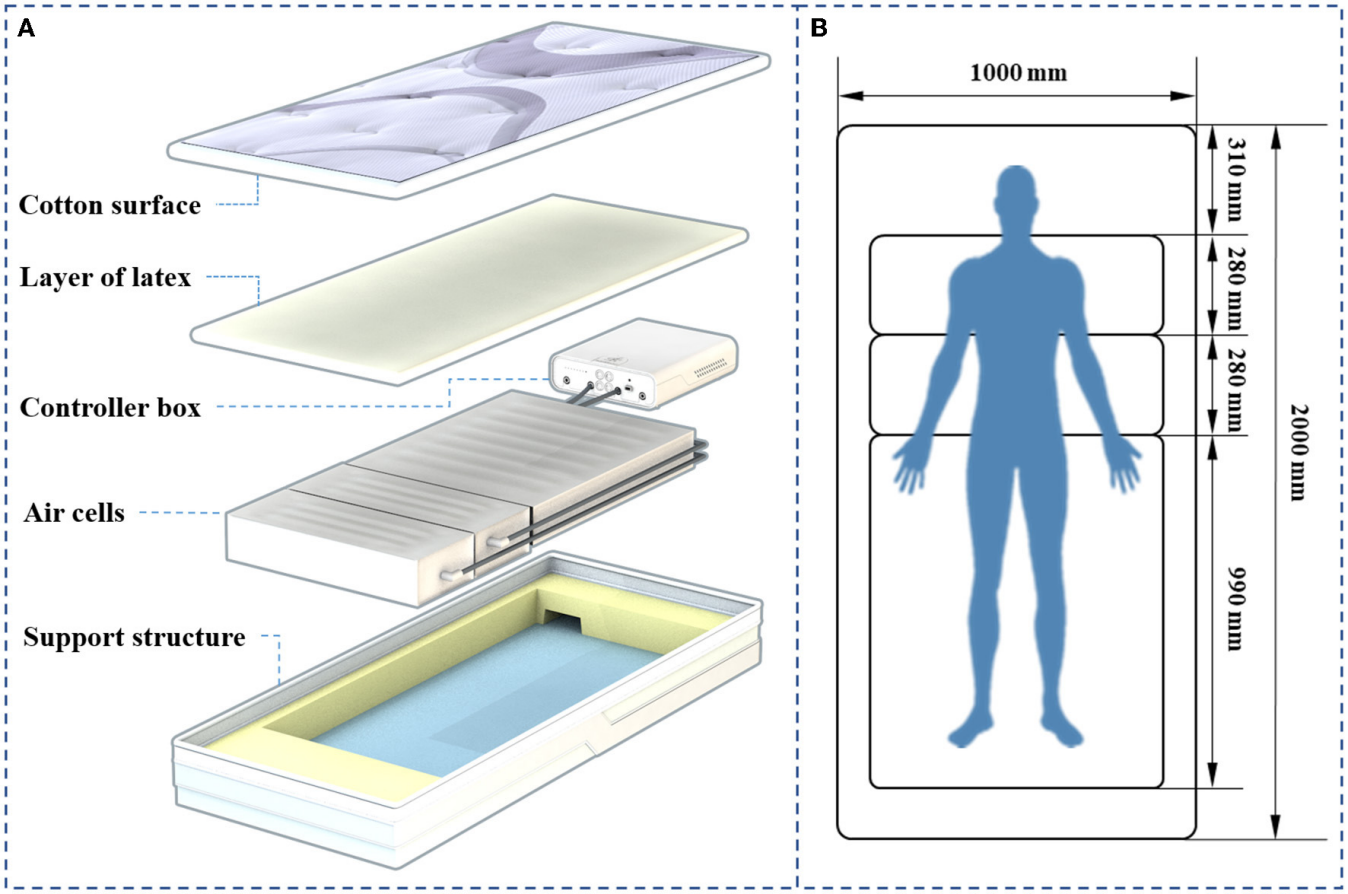
Structure and size of the auto air mattress. **(A)** The structure and materials of the auto air mattress. **(B)** The size of the zone.

In this study, two types of mattresses were used for the experiment, a specially designed adjustable zoned air mattress and an ordinary mattress, respectively. Both mattresses were ~100 cm in width and 200 cm in length. The adjustable zoned air mattress (hereinafter referred to as “auto air mattress”) contained three layers, as shown in Figure 1. The top layer was filled with polyester fibers with a cotton surface that was 3 cm thick. The latex layer was 5 cm thick, with a density of 70 kg/m^3^. The thickness of the air-cell layer was 16 cm. Its firmness could be controlled by the pressure of three separate, different-sized air cells (at the shoulder, waist, and hip). The internal pressures of these air cells could reach 4.5 kpa (the shoulder), 4.5 kpa (the waist), and 6.0 kpa (the leg), respectively, when they were filled. The ordinary mattress also had three layers: a cotton layer (4 cm), a spongy layer (1 cm), and a woven coir fiber layer (4 cm). The densities of the spongy and coir fiber layers were ~22 kg/m^3^ and 88 kg/m^3^, respectively.

**Figure 2:**
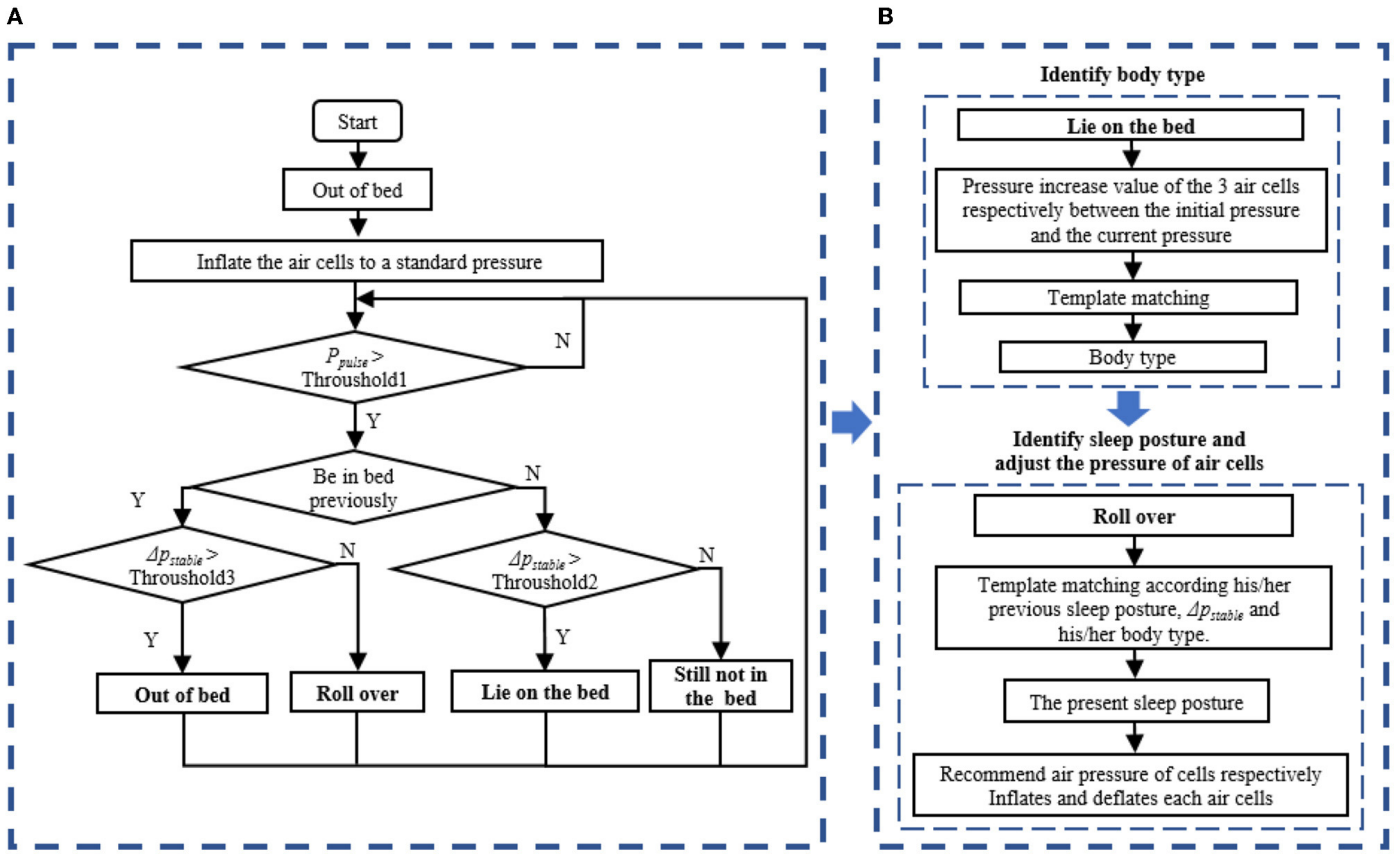
Schematic diagram of the control strategy. **(A)** Determine whether anyone is in bed and whether occurs a rolling action. **(B)** The process of pressure-adjusting when someone is in mattress and roll over is detected.

The design purpose of the auto air mattress is to provide comfortable pressure support and keep the spine in normal physiological curvature through real-time pressure regulation. The detection program and adjustment were performed every 5 min; the control strategy is shown in Figure 2.

The controller determines whether anyone is on the mattress and whether a rolling action occurs based on two pressure parameters: the sum of pressure pulses, p_pulse_, and the pressure difference between the current pressure and the mean stable pressure of the past 5 min, Δp_stable_. If the p_pulse_ was larger than the threshold, it means that some actions had happened. Following that action, Δp_stable_ was used to determine whether it was a rolling or out-of-bed action. If no one had previously occupied the bed, Δp_stable_ was used to confirm that someone was lying on it.

When someone lay on the mattress, the pressure on the air cells would increase. The sleeper's body type can be determined according to the increased value of the three air cells. If a rolling action was detected, sleep posture could be obtained according to his/her previous sleep posture, Δp_stable_, and his/her body type. We divided all the sleepers into six types, mainly by body mass index (BMI). Four sleep postures were defined, namely supine position, prone position, left lateral position, and right lateral position. The main objective of adjustment for supine and prone positions was a comfortable and uniform pressure distribution. We attempted to maintain the spine's natural curvature in the lateral position. The body types and sleep postures were both obtained by template matching. If someone was on the bed and a rolling action is detected, the controller would adjust the pressure of each air cell according to the body type and current sleep posture. For the rolling action, the speed of inflation or deflation would be relatively slow to ensure less stimulation to the sleeper.

### 2.3. PSG

The experiment was carried out in the sleep lab of Sun Yat-Sen University. The considerations and procedures were explained in detail to the participants before the experiments. The participants underwent PSG recordings using the Compumedics Profusion EEG Recording System with Neuvo Amplifier. Recorded data included 16 EEG channels (Fp1, Fp2, F3, F4, F7, F8, C3, C4, P3, P4, O1, O2, T3, T4, T5, and T6) placed based on the standard 10–20 system, electrooculography, electrocardiography (ECG), and electromyography. The EEG and ECG sampling frequencies were 500 and 1,250 Hz, respectively. The PSG recordings were scored (wake, NREM1, NREM2, NREM3, REM) in 30-s epochs by professional sleep technicians following the AASM standards.

### 2.4. Experimental design

Each participant was recorded for four consecutive nights. Four experimental sequences were designed to clarify the effects of experimental nights and mattresses. These four sequences are scheme I ordinary—ordinary—auto air—auto air (two males and three females), scheme II ordinary—auto air—ordinary—auto air (three males and two females), scheme III auto air—ordinary—auto air—ordinary (three males and two females), and scheme IV auto air—auto air—ordinary—ordinary (two males and three females). To show the multiple outcomes for individuals, the male participants were numbered 1–10 and the female participants were numbered 11–20. The numbering sequence is referred to as the scheme sequence. Due to equipment failure, the second night of female participant No. 14 (scheme II) was only recorded for nearly 5 h; therefore, we excluded that night from all statistics.

### 2.5. Assessment of sleep quality

#### 2.5.1. Subjective evaluation and objective evaluation based on sleep macrostructure

The subjective evaluation was carried out with a questionnaire survey after participants completed sleep monitoring. The self-rated indicators included total sleep time, sleep onset latency, number of awakenings during the night, awakening time, and sleep quality. Besides, self-reported sleep quality was scored from 1 to 5, with higher scores corresponding to a better status.

The objective sleep quality parameters are listed in Table 2. Seven recognized and commonly used evaluation indicators were evaluated, representing sleep continuity and sleep architecture (Mendonca et al., 2019).

**Table 2:** Objective sleep quality parameters.

**Feature description**	**Simplified formulae**
Sleep efficiency (%)	Total sleep time (TST)/time of recording (TRT)
	TRT = sleep latency + wake after sleep onset (WASO) + TST
Sleep latency (min)	Time from light out to the first sleep stage
N2 proportion (%)	∑ (N2 minutes)/TST
N3 proportion (%)	∑ (N3 minutes)/TST
REM proportion (%)	∑ (REM minutes)/TST
REM latency (min)	Time from the first sleep stage to the first REM stage
Wake after sleep onset (min)	Time of waking after sleep

#### 2.5.2. EEG spectral data processing and analysis

Segments with noticeable artifacts were excluded after a visual inspection. The power density value of each 30-s epoch was determined using the AR Burg method with 2-s Hamming windows. Following this, power density values were then averaged across delta (0.5 to ≤ 4 Hz), theta (>4 to ≤ 8 Hz), alpha (>8 to ≤ 11 Hz), sigma (>11 to ≤ 16 Hz), beta (>16 to ≤ 32 Hz), and gamma (>32 to ≤ 50 Hz) frequencies (Wang et al., 2022), and EEG relative power was calculated for each of the frequency bands as a function of the EEG total power (0.5 to ≤ 50 Hz) and averaged between 16 EEG channels. The relative power can reduce intra-individual variances and enhance the possibility of detecting changes within a spectrum (Herberger et al., 2020). However, we found in the results that there were also individual differences in power distribution, which will be discussed later in this manuscript.

#### 2.5.3. Extraction of heart rate variability features based on ECG

HRV time-domain and frequency-domain indexes were calculated to evaluate the cardiac ANS modulation during sleep. The difference threshold algorithm was used to detect the point of the R-peak, and we also manually checked these points for each beat to ensure accuracy (Yang et al., 2021). Based on this information, the RR interval was obtained, and the mean value of the RR interval (RRM) was calculated across 90-s epochs for use in the time domain. In addition, RMSSD (mean root square of deviation of adjacent RR interval time lengths), NN50 (the number of adjacent RR interval differences >50 ms), and pNN50 (the ratio of the number of adjacent RR interval time differences >50 ms to the total number of all RR intervals) were also calculated (Song et al., 2012). In the power spectral analysis, an autoregressive model and the Burg algorithm were adopted to estimate the power spectral density (Pichon et al., 2006). In this study, the powers in the frequency bands from 0.04 to 0.15 Hz and from 0.15 to 0.4 Hz were labeled very low-frequency power (LF) and high-frequency power (HF), respectively. The LF/HF ratio was also calculated.

### 2.6. Statistical analyses

The Mann–Whitney *U*-test was used to test for a significant difference in demographic characteristics between the sexes. The Wilcoxon Signed-Rank test was used to test the significant difference between the conditions of the two mattresses in subjective indices. To study the influence of the mattress and the experimental night in the group, we adopted the zero-mean method to eliminate the mean difference between individuals when the data of multiple participants were analyzed in a data set. The zero-mean method was adopted in sleep structure and efficiency analysis. Two-factor linear regression was used to analyze the influences of an experimental night and mattress on sleep efficiency and architecture. When we analyzed the data on 79 nights (except the second night of No. 14 because of equipment failure) for all participants, the influence of the order of the two mattresses was the same.

However, when we analyzed the EEG and ECG indicators, we did not add the data of multiple people into a data set for statistical analysis. This way, the large data fluctuations of a few participants may affect the entire statistical results of a group. The EEG and ECG indicators were analyzed in the individuals. Because the experimental night and the mattress were related factors for each participant, we conducted linear regression on these two factors separately. A significant effect was considered only when more than five participants of each sex showed the same significant difference, with no or just one opposite significant difference. This allowed us to assess the impact of various schemes on sleep performance across participants and between the sexes. However, we did not perform different schemes on one participant. We could not draw any conclusions about the scheme differences.

In addition, it was necessary to remove the epochs with noticeable artifacts that were heavily contaminated, and the proportions of these epochs in different stages were quite different. The effects of sleep stages on EEG and ECG parameters were observed. To maintain a participant's original sleep architecture, 20% of the original epochs (including polluted epochs and the wake period) were randomly retained in each sleep stage for every independent night. Random numbers were generated by random programs to ensure the authenticity of the data. Except for the N1 stage, the retained epochs were enough to perform statistical analysis for each sleep stage in the individuals.

All analyses were performed using IBM statistical software version 25.0. The level of significance was set at a *p*-value of <0.05.

## 3. Results

### 3.1. Subjective and objective sleep evaluation

After a rough examination of the results, we found that the influences of experimental nights and mattresses on the male and female participants differed. Therefore, male and female participants were analyzed separately.

**Figure 3:**
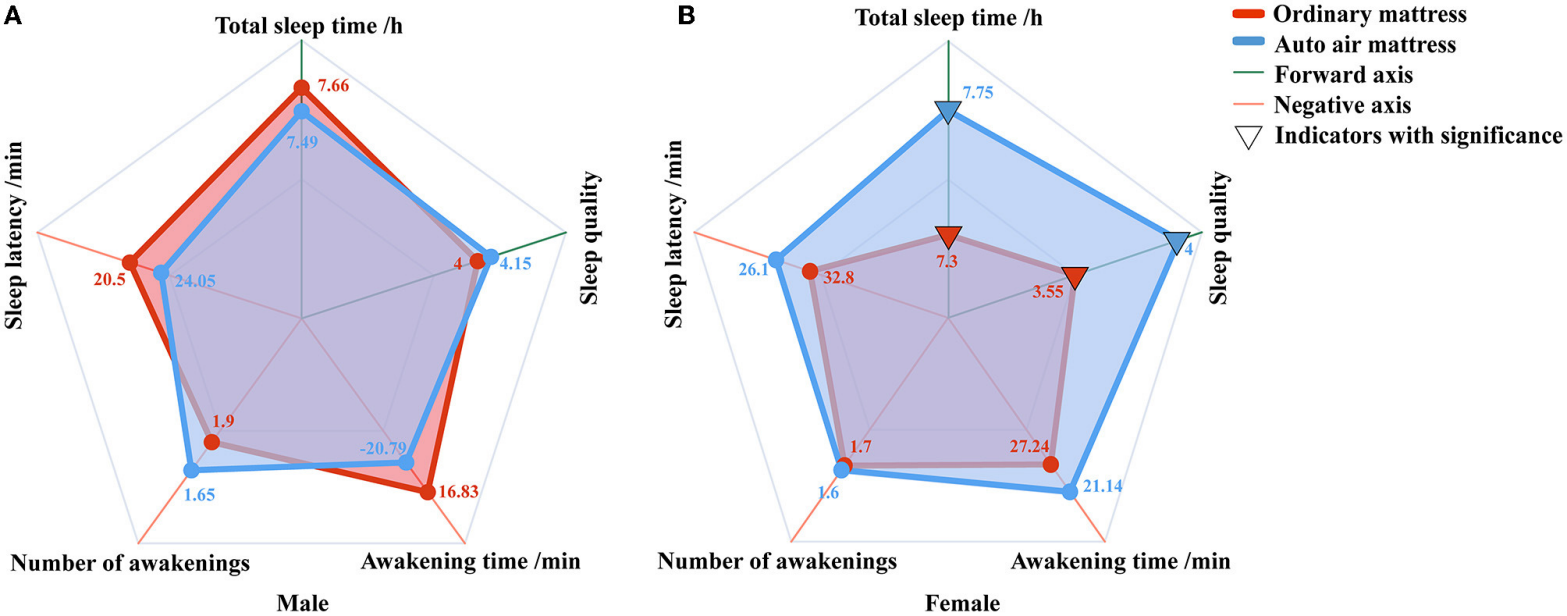
The subjective sleep evaluation of the participants. **(A)** The subjective sleep evaluation of male participants. **(B)** The subjective sleep evaluation of female participants.

The subjective evaluation of sleep is shown in Figure 3. No significant difference between the mattresses was observed in the researched subjective indexes for male participants. However, the total sleep time was significantly longer (*p* = 0.035), and the self-rated sleep quality was higher (*p* = 0.025) for female participants with the auto air mattress.

**Figure 4:**
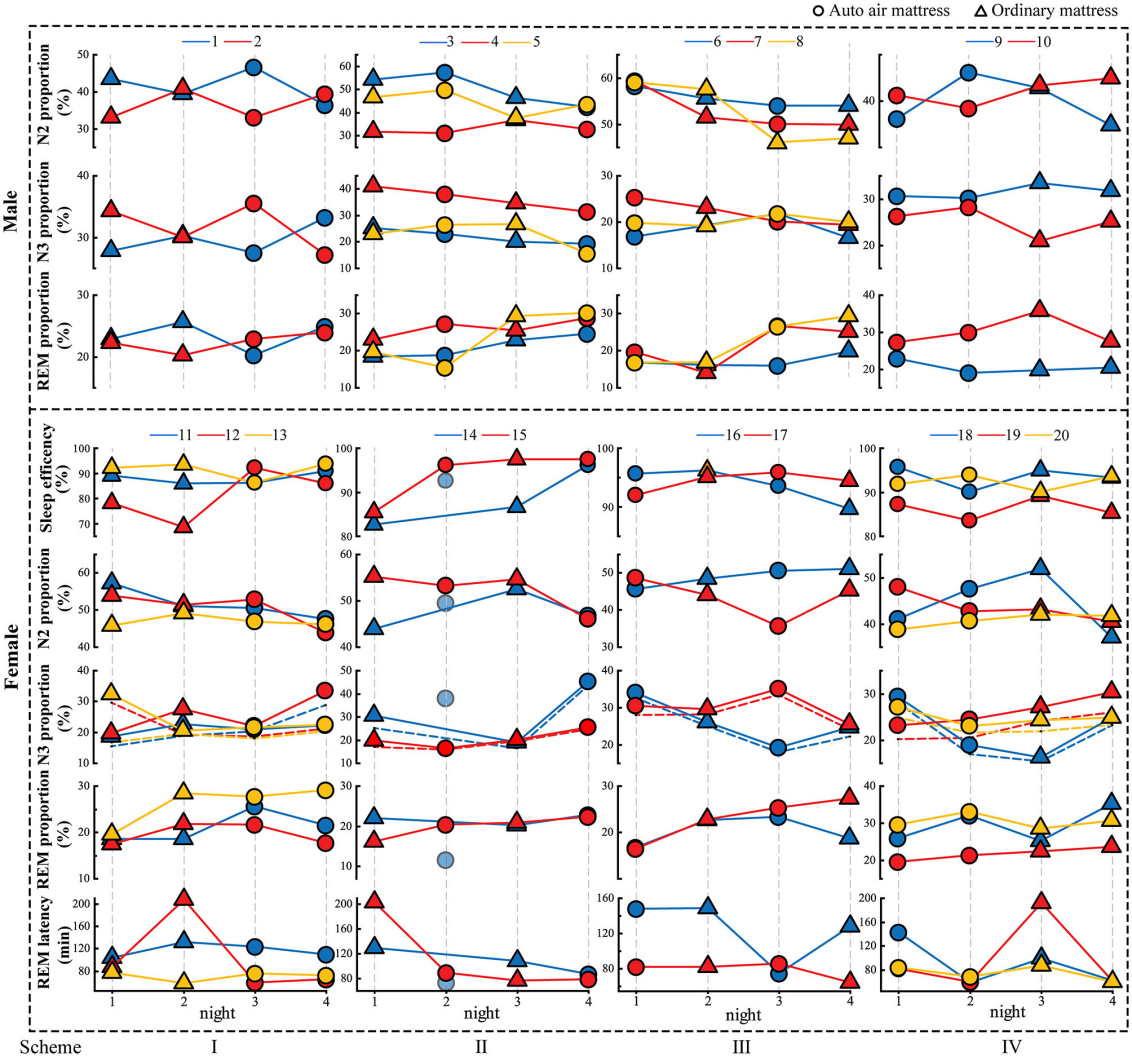
The objective evaluation parameters of the participants. The numbers 1 to 20 are the number of participants. The dashed line in N3% of female participants is the ratio of N3 sleep to the time of recording. The second night of No.14 was only recorded for nearly 5 h.

For objective sleep evaluation, we did not obtain a significant difference between the sexes in sleep efficiency or the proportions of N2, N3, and REM sleep. We were only concerned about the effects of mattresses and the experimental nights. Two-factor linear regression and the intra-individual zero-mean methods were used in this section. As shown in Table 3 and Figure 4, the male participants indicated a smaller response to different mattresses. However, our results indicated that auto-air mattresses significantly improved sleep efficiency for female participants. N2 proportion decreased, and the ratio of N3 sleep to time in bed (sleep efficiency ^*^ N3 proportion) increased with the auto air mattress in female participants, but its effect was just close to significance (sig = 0.086 and sig = 0.078, respectively), considering our samples were relatively small (20 nights for each mattress). We also checked the relationship between sleep efficiency and the ratio of N3 sleep to time in bed. There was a significant positive linear correlation between their zero-mean values. With an increase in experimental nights, both sexes indicated an increased REM proportion and a decreased N2 proportion; the N3 proportion decreased in the male participants, and REM latency decreased in the female participants. We also found that a large value of REM latency (more than 120 min) occurred more during the nights the participants slept on an ordinary mattress. Furthermore, there were no statistically significant results in the current results for sleep latency and wakefulness after sleep onset.

**Table 3:** Objective sleep evaluation of the whole night's sleep.

	**Male (10 participants)**	**Fsemale (10 participants)**
Sleep efficiency (%)	No significant impact	**Auto-air mattresses** and **experimental nights** both **improved sleep efficiency** •Mattress: *B* = 2.666, sig = 0.033 •Experimental nights: *B* = 1.119, sig = 0.043 •*R*^2^ = 0.207, DW = 2.485
Sleep latency (min)	No significant impact	
N2 proportion (%)	**Experimental nights decreased the N2 proportion** •Mattress: *B* = 0.605, sig = 0.596 •Experimental nights: *B* = −1.503, sig = 0.005 •*R*^2^ = 0.198, DW = 2.171	**Auto-air mattress** and **experimental nights** both **decreased N2 proportion**, but their effects were just close to significance •Mattress: *B* = −1.908, sig = 0.086 •Experimental nights: *B* = −0.892, sig = 0.071 •*R*^2^ = 0.156, DW = 2.413
N3 proportion (%)	**Experimental nights decreased the N3 proportion** •Mattress: *B* = −0.149, sig = 0.857 •Experimental nights: *B* = −0.943, sig = 0.014 •*R*^2^ = 0.152, DW = 2.065	**Auto-air mattresses** **increase the ratio of N3 sleep to time in bed**, but its effect was just close to significance •Mattress: *B* = 2.8, sig = 0.078 •Experimental nights: *B* = 0.5, sig = 0.499 •*R*^2^ = 0.095, DW = 2.155
REM proportion (%)	**Experimental nights increased the REM proportion** •Mattress: *B* = 0.225, sig = 0.879 •Experimental nights: *B* = 1.3771, sig = 0.010 •*R*^2^ = 0.165, DW = 1.166	**Experimental nights** **increased the REM** **proportion** •Mattress: *B* = 0.458, sig = 0.579 •Experimental nights: *B* = 1.384, sig <0.001 •*R*^2^ = 0.294, DW = 2.288
REM latency (min)	No significant impact	**Experimental nights** **decreased REM latency** •Mattress: *B* = 15.579, sig = 0.156 •Experimental nights: *B* = −11.784, sig = 0.018 •*R*^2^ = 0.188, DW = 2.313
WASO (min)	No significant impact	

The effects of mattresses and the experimental nights on objective sleep evaluation were presented. In this table, if the N2 proportion significantly decreases with an increase in experimental nights, we express this as “Experimental nights decreased the N2 proportion”, and if the sleep efficiency of Auto-air mattress is significantly higher than that of ordinary mattress, we express this as “Auto-air mattresses improved sleep efficiency”.

### 3.2. EEG relative power spectrum and heart rate parameters of the whole night's sleep

We randomly retained 20% of the original epochs in each sleep stage as a data set for each night. Linear regressions of the mattress and experimental nights were performed separately for every individual. As shown in Table 4, both sexes showed some influence of the mattress on heart rate parameters. Half of the male participants indicated a significant decrease in pHF, and half of the female participants showed lower NN50 with the auto air mattress, but one had an increased NN50.

**Table 4:** The results of EEG relative power and heart rate parameters.

**Indicators**		**Male (10 participants)**			**Female (10 participants)**		
		**Num**	**Increase/ decrease**	**Influence factor**	**Num**	**Increase/ decrease**	**Influence factor**
EEG relative power spectrum and heart rate parameters of the whole night's sleep							
Delta		5	Decrease	Experimental nights	No consistent and significant results		
Alpha		6	Increase	Experimental nights			
pHF		5	Decrease^1^	Auto air mattress			
NN50		No consistent and significant results			5	Decrease^1^	Auto air mattress
RRM		5	Increase^1^	Experimental nights	7	Increase^1^	Experimental nights
EEG relative power spectrum and heart rate parameters of various sleep stages							
N2	Alpha	No consistent and significant results			5	Increase^1^	Auto air mattress
		RRM			7	Increase^2^	Experimental nights
N3	NN50	6	Decrease^1^	Auto air mattress	5	Decrease	Auto air mattress
	pNN50	5	Decrease	Auto air mattress	4	Decrease	Auto air mattress
	RRM	7	Increase^1^	Experimental nights	5	Increase^1^	Experimental nights
	RMSSD	3	Decrease	Auto air mattress	4	Decrease	Auto air mattress
	pLF	3	Decrease	Auto air mattress	3	Decrease	Auto air mattress
	pHF	3	decrease^1^	Auto air mattress	3	Decrease	Auto air mattress
	LH	No consistent and significant results			3	Decrease	Auto air mattress
REM	Gamma	No consistent and significant results			5	Increase^1^	Experimental nights
	RRM	5	Increase^1^	Experimental nights	7	Increase^1^	Experimental nights

The “Num” represents the number of participants with significance. “^1^” and “^2^” represents the number of participants who showed the opposite result, and more details are shown in Supplementary Tables 1, 2.

**Figure 5:**
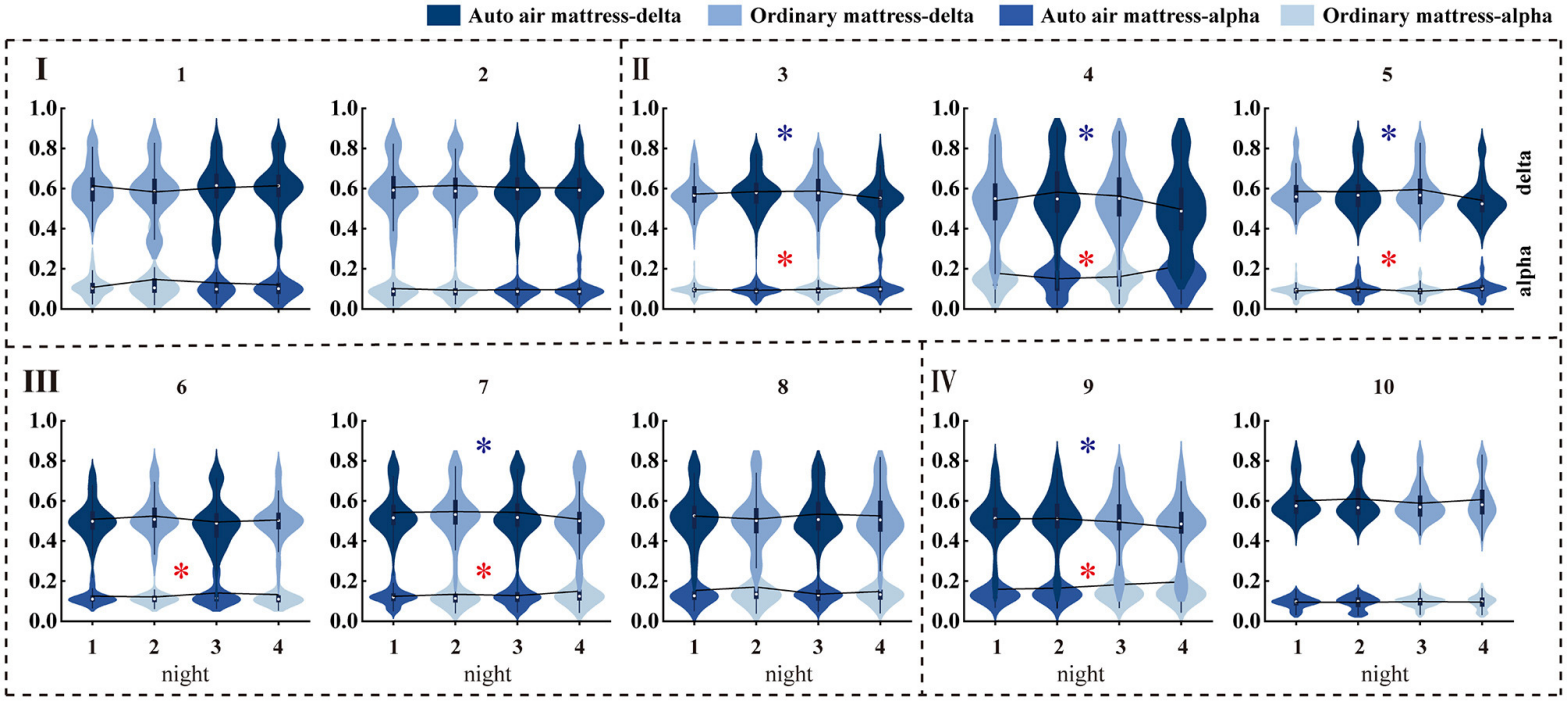
The delta and alpha power distributions of each male participant throughout the night. Blue star and red star indicate that delta power significantly decreases and alpha power increases with the experimental nights, respectively.

The male participants showed a more significant and coincident correlation with experimental nights. Half of the male participants indicated a delta power decrease, and six out of 10 male participants showed an alpha increase. The delta power distribution of each male participant throughout the night is shown in Figure 5. There were no consistent findings of significance in female participants for the whole night's EEG samples. Significant increases in RRM were obtained in half of the male participants and seven out of 10 female participants, and one participant of each sex showed the opposite significance (more details are shown in Supplementary Table 1).

### 3.3. Influence in various sleep stages

EEG relative power and heart rate-related indexes were statistically analyzed for each sleep stage. The N1 stage was not included in these statistics due to individuals' lack of statistical samples. Unlike the results of the whole night, female participants indicated more significant results in separate sleep stages, in both the influence of the mattress and experimental nights.

**Figure 6:**
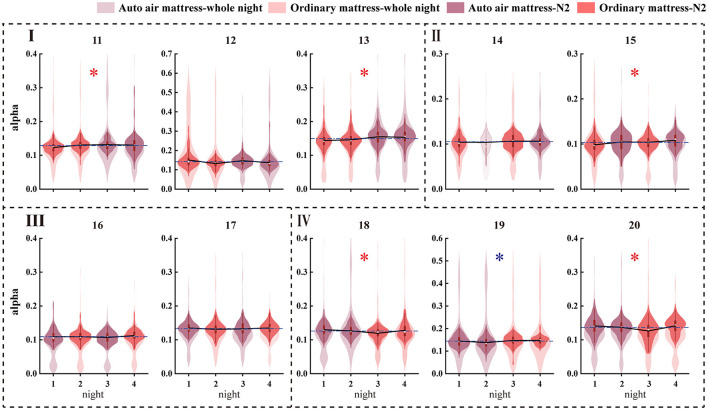
The alpha power distribution of the whole night and the N2 sleep of the female participants. Red star and blue star indicate that the auto air mattress increases relative alpha power and decreases alpha relative power, respectively.

As shown in Table 4, half of the female participants showed a significant alpha increase during N2 sleep for EEG relative power. Half of the participants indicated an increased gamma band during REM sleep. However, both had one oppositely significant result. No consistently significant result for male participants was indicated during N2, N3, and REM sleep. The alpha power distribution of the whole night and the N2 sleep of female participants are shown in Figure 6.

Air-filled auto mattresses significantly weakened ANS activity during N3 sleep. The effect was more consistent for the female participants. The decrease in NN50, pNN50, and RMSSD can be obtained in both sexes. We also found decreased pLF, pHF, and LH in the female participants. There was no opposite significant result in the female participants. In addition, experimental nights increased RRM during N3 and REM sleep in both sexes and N2 sleep in the female participants. In addition, individual differences can be observed in these influences. For example, among the male particpants, participant Nos. 3, 4, and 8 tended to be more sensitive to the mattress. Participant Nos.4 and 19 tended to have significantly opposite results on the influence of the experimental night on RRM. Participant No. 19 also indicated an opposite result in relative alpha power during N2 sleep. We also noticed that, although some decreased heart rate parameters were obtained for all other female participants during N3 sleep with auto-air mattresses, both female participants in Scheme II did not indicate these significant effects. Participant No. 15 even showed an increased NN50 with auto-air mattresses in the whole night statistics (more details are presented in Supplementary Table 2).

## 4. Discussion

The initial purpose of this study was to determine whether a real-time ergonomically adjusted mattress improves sleep and which parameters can be affected by mattresses. However, according to our results, the experimental night was also an important influencing factor for both the male and female participants. The female participants tended to be more sensitive to mattresses, and the auto-air mattress improved their sleep efficiency. The female participants could also perceive this improvement, which is reflected in subjective sleep quality improvement and duration lengthening. Most female participants indicated decreased ANS activity during N3 sleep. Approximately half of the male participants also indicated decreased ANS activity, but its effect was less significant. Experimental nights had some of the same significant effects on both sexes. Reduced heart rate, increased REM proportion, and decreased N2 proportion were obtained with the increase in experimental nights.

First, the basic parameter differences among individuals in the results were unexpected, as shown in Figure 4. This individual difference occurs on the premise that there was no significant difference between the male and female participants in these parameters. Studies have found that the transcriptional repressor DEC2 is related to total sleep time in mammals (He et al., 2009). The polymorphism in the circadian clock gene PERIOD3 (PER3) could influence the sleep homeostasis of the individual (Viola et al., 2007), which may provide some explanations for this inter-individual difference (Funato and Yanagisawa, 2022). Zhang et al. (2022a) provided an umbrella review of PSG parameters in 27 neuropsychiatric diseases, suggesting that each disease may relate to a specific sleep profile. Although we enrolled healthy participants in this study, we also had a reason to doubt whether this individual consistency difference is a personal sleep trait and whether it is related to long-term psychological health.

We found that the female participants tended to be more sensitive to mattresses. Previous research has also shown that women pay more attention to sleep over other activities at night (Ruggiero et al., 2019). They made greater demands of a sleep environment (Bjorvatn et al., 2017). Differences in body shapes between the sexes require specific pressure designs in mattresses (Hu et al., 2019). The participants mainly slept in a supine position limited by PSG recording equipment during the experiment. In this situation, a comfortable and uniform pressure distribution is our objective. The participants reported that the air mattress was softer than the control mattress. Our previous studies indicated that MDD patients showed a consistent enhancement of functional connectivity (Lian et al., 2021; Song et al., 2022; Zhang et al., 2022b), and the female participants exhibited stronger EEG functional connectivity than the male participants (Liao et al., 2019). We are aware that female patients are more likely to suffer from depression (Eid et al., 2019; Gao et al., 2020b) and that patients with depression are more sensitive to the outside environment. Through this study, we observed the possibility of improving mental conditions through the design of mattresses or sleeping environments.

The effect of auto-air mattresses is reflected in higher sleep efficiency in the female participants and decreased ANS activity during N3 sleep. First, the control mattress is also relatively comfortable, which was confirmed by all participants. We found that increased sleep efficiency and reduced ANS activity associated with comfortable or ergonomically adaptable mattresses in this study have also been found in previous research (Lee and Park, 2006; Kuo et al., 2013). We further refined the results, which are mutually consistent in different aspects. We found the main decrease in cardiovascular sympathetic modulation occurred during N3 sleep, and the decreased NN50, pNN50, and RMSSD may be the reason for the improved sleep efficiency. Referring to the review of Zhang et al. (2022a), 21 types of neuropsychiatric diseases indicated at least weak evidence of decreased sleep efficiency. Thus, the above evidence suggested that auto-air mattresses had a positive effect on sleep performance.

The influence of experimental nights may be a new finding, and our results should be detailed supplements to the first night effect (Agnew et al., 1966). We used the same experimental conditions for each night; therefore, the impact of the experimental nights should be due to psychological factors. In the male participants, we observed a decreased N3 proportion and delta relative power. Delta power was considered to indicate sleep pressure. Our results indicated that the sleep pressure of the male participants decreased with the increase in nights. Reduced heart rate and N2 proportion and increased REM sleep were also found with the increase in experimental nights. Smagula et al. (2015) reported that older men with depressive symptoms spent more time in N2 but less time in REM sleep. Therefore, it is reasonable to speculate that, with the increase in experimental nights, sleep develops in the opposite direction of depression. Moreover, we also noticed that air-filled auto mattresses decreased the proportion of N2 sleep in the female participants. Moreover, these findings suggest that the architecture of a person's sleep can be altered, further suggesting the possibility of improving mental health by improving sleep psychology, for example, maintaining a consistent sleeping environment.

There are limitations to this study. First, we did not implement different schemes for the same participants. Although we observed some differences in the results between the schemes, we could not determine whether they were due to the scheme or just an individual difference. Second, each participant only participated in a four-night PSG experiment in this study. The habit of using a certain mattress may affect the results. Moreover, we did not ensure sleep reliability by sleep diary and activity before PSG instead, we used the subjective method (i.e., questionnaires and conversations). In addition, the participants in this study were healthy undergraduate and graduate students with a narrow range of age and BMI. Considering the above limitations, we intend to use this adjustable mattress to conduct long-term sleep experiments with sleepers with different BMIs, preferences, and ages.

## 5. Conclusion

In summary, both the sleep mattress and the experimental night affected sleep performance. The female participants tended to be more sensitive to the mattress. This study provided evidence and relevant parameters concerning their influences. Several of the findings may be related to the psychology of sleep. Therefore, our results may improve mental health through the design of the sleep environment. In addition, we will further research the adjustment strategies to cater to different participants' sleep preferences and habits.

## Data availability statement

The raw data supporting the conclusions of this article will be made available by the authors, without undue reservation.

## Ethics statement

The studies involving human participants were reviewed and approved by the Ethics Committee of Guangdong 999 Brain Hospital. The patients/participants provided their written informed consent to participate in this study.

## Author contributions

YW and YZhu: conceptualization, methodology, and writing—original draft. YZho: investigation and data curation. XY, HL, and LR: visualization and writing—review and editing. HL and YL: supervision and validation. All authors contributed to the article and approved the submitted version.
