# Supplementary material for: Investigating the influence of an adjustable zoned air mattress on sleep: a multinight polysomnography study
Source: Front Neurosci. 2023 Apr 20;17:1160805. doi: 10.3389/fnins.2023.1160805 (PMC10156966; doi:10.3389/fnins.2023.1160805)
Supplement: Supplementary file 1 [file Data_Sheet_1.docx]

Supplementary Material

Multinight polysomnography study on the influence of an adjustable zoned air mattress

Yu Wei *, Yongpeng Zhu *, Yihan Zhou, Xiaokang Yu, Huiping Lin, Lijun Ruan

*** Correspondence:** Yuxi Luo: [luoyuc@163.com](mailto:luoyuc@163.com), Hua Lei: yxzx-leih@derucci.net

# Supplementary Tables

**Table 1.** EEG relative power spectrum and heart rate parameters of the whole night sleep

|  | Males (10 participants) | Females (10 participants) |
| --- | --- | --- |
| EEG relative power spectrum of the whole night sleep | | |
| Twenty percent of the original epochs in each sleep stage for every independent night | **Delta power of 5 participants** significantly **decreased with the experimental nights**  They are Scheme II: No. 3, 4 and 5,  scheme III: No. 7, scheme IV: No. 9  No opposite result. | No consistent and significant results.  In fact, there is almost no frequency had more than 5 significances. |
|  | **Alpha power of 6 participants** significantly **increased with the experimental nights**  They are scheme II: No. 3, 4 and 5, scheme III: No. 6 and 7, scheme IV: No. 9  No opposite result. |  |
| Heart rate parameters of the whole night sleep | | |
| Twenty percent of the original epochs in each sleep stage for every independent night | **Five participants** indicated **auto air mattress** significantly **decreased pHF.**  They are Scheme I: No. 1, Scheme II: No. 3 and 4,  Scheme III: No. 8, Scheme IV: No. 10  No.6 of Scheme III show the opposite result. | **Five participants** indicated **auto air mattress** significantly **decreased NN50.**  They are Scheme I: No. 11, 12 and 13  Scheme III: No. 16 and 17  No.15 of Scheme II show the opposite result. |
|  | **RRM of 5 participants** significantly **increased with the experimental nights**  They are Scheme II: No. 5, Scheme III: No. 6, 7 and 8, Scheme IV: No. 10  No.4 of Scheme II show the opposite result. | **RRM of 7 participants** significantly **increased with the experimental nights**  They are Scheme I: No. 12 and 13, Scheme II: No. 14 and 15, Scheme III: No. 16 and 18, Scheme IV: No. 20  No.19 of Scheme IV show the opposite result**.** |

**Table 2.** EEG relative power spectrum and heart rate parameters of various sleep stages

|  | Males (10 participants) | Females (10 participants) |
| --- | --- | --- |
| EEG relative power spectrum | | |
| N2 | No consistent significant differences | **Five participants** indicated **auto air mattress increased alpha relative power**  They are Scheme I: No. 11 and 13, Scheme II: No. 15,  Scheme III: No. 18, Scheme IV: No. 20  No.19 of Scheme IV show the opposite result**.** |
| N3 | No consistent significant differences | |
| REM | No consistent significant differences | **Five participants** indicated **gamma power** increased **with experimental nights**  They are Scheme I: No. 11, Scheme II: No. 14,  Scheme III: No. 17, Scheme IV: No. 20  No.13 of Scheme I showed the opposite result**.** |
| Heart rate parameters | | |
| N2 | No consistent significant differences | **RRM of 7 participants** significantly **increased with the experimental nights**  They are Scheme I: No. 12 and 13, Scheme II: No. 14 and 15, Scheme III: No. 16 and 17, Scheme IV: No. 20  No.11 of Scheme I and No.19 of Scheme IV show the opposite result**.** |
| N3 | **With auto air mattress** | |
|  | **NN50 of 6 participants decreased**  They are Scheme I: No. 2, Scheme II: No.3, 4 and 5, Scheme III: No. 8, Scheme IV: No. 10  **pNN50 of 5 participant decreased**  They are Scheme II: No.3, 4 and 5, Scheme III: No. 8, Scheme IV: No. 10  **RMSSD of 3 participants decreased**  Scheme II: No.3 and 4, Scheme III: No. 8  Three **participant**s indicated decreased pLF or pHF  They are Scheme II: No.3, 4, Scheme III: No. 8  Only No. 6 of Scheme III had increase in NN50 and pHF, but **decreased** LH | Scheme I:  No.11 had a **decrease** in NN50  No.12 had **decreased** RMSSD, NN50, pNN50, pLF, pHF and LH  No.13 had **decreased** RMSSD, NN50 and pNN50, |
|  |  | Scheme II:  **Not any significant difference about mattress** in **participant** No.14 and 15. |
|  |  | Scheme III:  Both No.16 and No.17 had **decreased** RMSSD, NN50, pNN50, pLF and pHF, in addition, No.17 LH decreased |
|  |  | Scheme IV:  No.18 had **decreased** RMSSD and pHF  No.19 had **decreased** NN50 and pNN50  No. 20 had **decreased** pLF and LH. |
|  |  | **Not any increased results in RMSSD, NN50, pNN50, pLF, pHF or LH with air auto mattress.** |
|  | No consistent significant differences in RRM about mattress | |
|  | **With the increase of experimental nights** | |
|  | **RRM of 7 participants** significantly **increased**  They are Scheme I: No. 1 and 2, Scheme II: No. 3 and 5, Scheme III: No. 6 and 8, Scheme IV: No. 10  No.4 of Scheme II show the opposite result. | **RRM of 5 participants** significantly **increased**  They are Scheme I: No. 11, 12 and 13, Scheme II: No. 14, Scheme IV: No. 20  No.19 of Scheme IV show the opposite result**.** |
| REM | **RRM of 5 participants** significantly **increased with Experimental night**  They are Scheme II: No. 3, Scheme III: No. 6,7 and 8, Scheme IV: No. 10.  No.4 of Scheme II show the opposite result. | **RRM of 7 participants** significantly **increased with Experimental night**  They are Scheme I: No. 11, 12 and 13, Scheme II: No. 14 and 15, Scheme IV: No. 18 and 20  No.19 of Scheme IV show the opposite result**.** |
